# Glutamine and cystine-enriched diets modulate aquaporins gene expression in the small intestine of piglets

**DOI:** 10.1371/journal.pone.0245739

**Published:** 2021-01-19

**Authors:** Inês Vieira da Silva, Bárbara P. Soares, Catarina Pimpão, Rui M. A. Pinto, Teresa Costa, João P. B. Freire, Etienne Corrent, Tristan Chalvon-Demersay, José A. M. Prates, Paula A. Lopes, Graça Soveral

**Affiliations:** 1 Research Institute for Medicines (iMed.ULisboa), Faculdade de Farmácia, Universidade de Lisboa, Lisbon, Portugal; 2 Dept. Bioquímica e Biologia Humana, Faculdade de Farmácia, Universidade de Lisboa, Lisboa, Portugal; 3 JCS, Laboratório de Análises Clínicas Dr. Joaquim Chaves, Algés, Portugal; 4 Indukern Portugal, Lda., Centro Empresarial Sintra Estoril II, Sintra, Portugal; 5 LEAF—Linking Engineering, Agriculture and Food, Departamento de Ciências e Engenharia de Biossistemas, Instituto Superior de Agronomia (ISA), Universidade de Lisboa, Lisboa, Portugal; 6 Ajinomoto Animal Nutrition Europe, Paris, France; 7 CIISA—Centro de Investigação Interdisciplinar em Sanidade Animal, Faculdade de Medicina Veterinária, Universidade de Lisboa, Lisboa, Portugal; Institute of Biomedicine of Seville-IBiS, SPAIN

## Abstract

The regulation of glycerol permeability in the gastrointestinal tract is crucial to control fat deposition, lipolysis and gluconeogenesis. Knowing that the amino acid glutamine is a physiological regulator of gluconeogenesis, whereas cystine promotes adiposity, herein we investigated the effects of dietary supplementation with glutamine and cystine on the serum biochemical parameters of piglets fed on amino acid-enriched diets, as well as on the transcriptional profile of membrane water and glycerol channels aquaporins (AQPs) in the ileum portion of the small intestine and its impact on intestinal permeability. Twenty male piglets with an initial body weight of 8.8 ± 0.89 kg were allocated to four dietary treatments (n = 5) and received, during a four week-period, a basal diet without supplementation (control) or supplemented with 8 kg/ton of glutamine (Gln), cystine (Cys) or the combination of the two amino acids in equal proportions (Gln + Cys). Most biochemical parameters were found improved in piglets fed Gln and Cys diet. mRNA levels of *AQP3* were found predominant over the others. Both amino acids, individually or combined, were responsible for a consistent downregulation of *AQP1*, *AQP7* and *AQP10*, without impacting on water permeability. Conversely, Cys enriched diet upregulated *AQP3* enhancing basolateral membranes glycerol permeability and downregulating glycerol kinase (*GK*) of intestinal cells. Altogether, our data reveal that amino acids dietary supplementation can modulate intestinal AQPs expression and unveil AQP3 as a promising target for adipogenesis regulation.

## Introduction

Over the past decade, there has been increasing recognition of an association between disrupted intestinal barrier function and disease pathogenesis, in both animals and humans [1]. Recent studies have demonstrated that obesity, type 2 diabetes, inflammation and metabolic complications, such as dyslipidemia, are associated with intestinal barrier dysfunction and exacerbated by a lipid challenge [2, 3], suggesting that modulation of intestinal barrier permeability or personalized nutrition therapy may represent novel therapeutic approaches [4].

Glycerol concentration in plasma is determined primarily by adipose tissue lipolysis, but also by the variable amount reabsorbed in kidney tubules and gastrointestinal tract. In the interprandial state, starvation and exercise, glycerol from lipolysis represents an important substrate for gluconeogenesis together with lactate, pyruvate and some amino acids, as the case of glutamine [5]. Amino acids are building blocks of protein but also have many other important functions within the body, such as cell proliferation, immunity modulation, food intake control and energy provision [6]. While glutamine acts both as substrate and regulator of gluconeogenesis [5, 7, 8], cystine promotes adiposity by increasing visceral fat accumulation and lowering insulin sensitivity in conjunction with changes in genes expression involved in lipid and glucose metabolism [9]. In fasting conditions, triacylglycerol hydrolysis in the adipose tissue results in non-esterified fatty acids and glycerol, the latter being used for gluconeogenesis in the liver to assure normoglycemia. These metabolic events occurring exclusively in intracellular compartments depend on glycerol transport across different tissues [10]. Glycerol diffusion across cell membranes is facilitated by aquaglyceroporins, a subfamily of aquaporin water channels (AQPs) [11, 12] which are divided into three major sub-classes, according to their primary sequence and permeability features: (1) classical or orthodox aquaporins, considered primarily selective to water (AQP0, AQP1, AQP2, AQP4, AQP5, AQP6 and AQP8); (2) aquaglyceroporins, which have a less-constricted pore allowing permeation of small molecules like glycerol (AQP3, AQP7, AQP9 and AQP10); and (3) super-aquaporins or subcellular aquaporins, with intracellular localization and still undefined selectivity (AQP11 and AQP12) [13, 14]. Although the precise localization of AQPs in human intestine membranes is still unclear, their function as water channels in both healthy and pathological gastrointestinal related conditions, such as infectious diarrhea, inflammatory bowel disease and celiac disease, is well documented [15–18].

In this study, the expression and function of the water channel *AQP1* and the glycerol channels *AQP3*, *AQP7*, *AQP9* and *AQP10* involved in volume regulation and energy homeostasis respectively, were investigated in the ileum portion of the small intestine of piglets. The pig is rapidly emerging as a biomedical model for energy metabolism and human obesity because of their similar metabolic features, cardiovascular systems, and proportional organ sizes [19]. To ascertain the effect of amino acids, glutamine and cystine-enriched diets, we firstly characterised the general metabolic state of piglets followed by the modulation of intestinal *AQPs* gene expression and activity. To understand the mechanism of action, we also determined the transcriptional profile of glycerol kinase (*GK*), the key enzyme in the regulation of glycerol uptake and metabolism [20] and the intracellular mediators involved in the regulation of *AQPs* expression, focusing on the phosphatidylinositol**-**3-kinase/protein kinase B (*PI3K*/*Akt*) and mammalian target of rapamycin (*mTOR*) transduction signals [21, 22]. Finally, the impact of glutamine and cystine-enriched diets on intestinal permeability of piglets was evaluated using isolated membranes from the ileum portion of the small intestine.

## Material and methods

### Animals and diets

All experimental procedures were reviewed and approved by the Animal Care Committee of Instituto Superior de Agronomia (ISA), Universidade de Lisboa (Lisbon, Portugal) and authorized by the National Veterinary Authority (Direcção Geral de Alimentação e Veterinária (Lisbon, Portugal), following the appropriate European Union guidelines (2010/63/EU Directive) to minimize animals suffering. Paula A. Lopes and José A. M. Prates hold a FELASA C grade, which enables them to design and conduct animal experimentation in the European Union.

This study was performed at the Animal Sector of the Engineer Bio-Systems, Instituto Superior de Agronomia, ISA, Universidade de Lisboa (Lisbon, Portugal). Before the beginning of the experiment, all piglets were subjected to the same management and received the conventional commercial starter diet (INICEAL PLASMA G), dosing 19.5% of crude protein (CP), 1.40% of lysine and 10.4 MJ/kg NE, manufactured by Eurocereal (Comercialização de Produtos Agro-Pecuários, SA, Portugal). Twenty male piglets, weaned at 4 weeks, with an initial average weight of 8.8 ± 0.89 kg (mean ± SD) from a F1 cross between the Large White and Landrace breeds were allocated to four groups (5 pigs *per* diet) and received during a 4 week-period a basal diet with 27.5% of crude protein (NRC, 1998) and without supplementation (Control group) or supplemented with 8 kg/ton of glutamine (Gln group), cystine (Cys group) or the combination of the two amino acids in equal quantity (Gln + Cys group). The ingredients of the experimental diets, together with their amino acid composition, are presented in Table 1. Throughout the experiment, piglets were weighed weekly before feeding. By the end of the experimental period, animals were sacrificed by cutting the carotid artery and external jugular veins after stunning by electronarcosis, according to EU legislation [23]. Blood samples were collected from the jugular vein and centrifuged at 1500 *g* for 15 min to obtain serum. Samples from the ileum portion of the small intestine (60 cm before the ileo-caecal valve) were removed, washed with saline, flash-frozen in liquid nitrogen and stored at -80°C for subsequent analysis.

**Table 1:** Ingredients (g.kg^-1^) composition of the experimental diets.

	Control	Gln	Cys	Gln + Cys
Corn	140	140	140	140
Wheat	356	356	356	356
Sweet dry whey	150	150	150	150
Soybean meal (48)	270	270	270	270
Soybean oil	40	40	40	40
L-Lys	5.3	5.3	5.3	5.3
L-Thr	2.5	2.5	2.5	2.5
DL-Met	3.2	3.2	3.2	3.2
L-Thp	0.8	0.8	0.8	0.8
L-Val	1.8	1.8	1.8	1.8
L-Gln	0.0	8.0	0.0	4.0
L-Cys	0.0	0.0	8.0	4.0
CaCO_2_	7.0	7.0	7.0	7.0
Dicalcium phosphate	15	15	15	15
NaCl	3.0	3.0	3.0	3.0
Vitamin trace mineral mix^(1)^	5.0	5.0	5.0	5.0

^(1)^Vitamin and trace mineral supplied *per* kilogram of diet–Vit. A: 25 000 IU; Vit. D3: 2000 IU; Vit. E: 20 IU; Vit C: 200 mg; Vit. B1: 1.5 mg; Vit. B2: 5 mg; Vit. B3: 30 mg, Vit. B5: 15 mg; Vit. B6: 2.5 mg; Vit. B9: 0.5 mg; Vit. B12: 0.03 mg; Vit. K3: 1 mg; Vit. H2: 80 mg; choline (chloride): 300 mg; I: 1 mg as potassium iodate; Mn: 50 mg as manganese (oxide); Fe: 120 mg as ferrous carbonate; Zn: 140 mg as zinc (oxide); Cu: 160 mg as copper sulphate; Se: 0.3 mg as sodium selenite; Co: 0.5 mg as cobalt carbonate.

### Serum biochemical profile and redox status

Glucose, urea, creatinine, total cholesterol, HDL-cholesterol, LDL-cholesterol, triacylglycerols (TAG), total protein, phospholipids, alanine aminotransferase (ALT), aspartate aminotransferase (AST), alkaline phosphatase (ALP) and γ-glutamiltransferase (GGT) were analysed in serum using diagnostic kits (Roche Diagnostics, Mannheim, Germany) and a Modular Hitachi Analytical System (Roche Diagnostics). VLDL-cholesterol and total lipids were calculated as described by Friedewald et al. [24] and Covaci et al. [25], respectively. The quantitative colorimetric determinations of total antioxidant capacity (DTAC-100) and glutathione peroxidase activity (EGPX-100) were measured using commercial kits (BioAssay Systems, Hayward, CA, USA).

### RNA isolation and cDNA synthesis

The RNA isolation was performed in the ileum samples by homogenization with a TissueLyser II (Qiagen, Hilden, Germany) using TRIzol® Reagent (Invitrogen, Barcelona, Spain) and extraction was performed according to the manufacturer’s instructions. For first strand cDNA synthesis, constant amounts of 1 μg of total RNA with quality ratios 260/280 and 260/230 between 1.8–2.2 (NanoDrop1 ND-2000c, ThermoFisher Scientific, Waltham, USA) were reverse transcribed in a 20 μl final volume using NZYFirst-strand cDNA synthesis Kit (NZYtech, Lisbon, Portugal), according to the manufacturer’s protocol.

### Real-time quantitative PCR (RT-qPCR)

The transcript levels for *AQP1*, *AQP3*, *AQP7*, *AQP9*, *AQP10*, *GK*, *mTOR* and *PIK3CG* and housekeeping, β-actin (*ACTB*) and hypoxanthine phosphoribosyltransferase 1 (*HPRT1*) genes were quantified in the ileum portion of small intestine samples by real-time quantitative PCR in a CFX96™ Real-Time PCR Detection System C1000 (BioRad, CA, USA). RT-qPCR was performed using TaqMan™ Universal Master Mix II with UNG (Life Technologies, CA, USA) and following *Sus scrofa* specific predesigned TaqMan™ Gene Expression Assays (primers FWD, REV and FAM-labelled probe; Life Technologies, CA, USA): *AQP1*, Ss03385017_u1; *AQP3*, Ss03389620_m1; *AQP7*, Ss03389863_m1; *AQP9*, Ss03389741_m1; *AQP10*, Ss03374224_m1; *GK*, Ss03818509_m1; *mTOR*, Ss03377427_u1; *PIK3CG*, Ss03392908_u1; *ACTB*, Ss03376081_u1 and *HPRT1*, Ss03388274_m1. The characterisation of the selected genes used in real-time quantitative PCR is described in Table 2. Probes were designed to hybridise between exons to ensure the detection of the corresponding transcript avoiding genomic DNA amplification. The cDNA was amplified at the following conditions: 95°C for 10 min, followed by 45 cycles of 15 seconds at 95°C and 1 min at 59°C, using the TaqMan® Universal PCR Master Mix (Applied Biosystems, Foster City, CA, USA). The reaction volume of 20 μL was prepared using 10 μL of TaqMan Master Mix, 1 μL of Taqman Assay and 9 μL of cDNA template diluted to 1:8 in molecular biology grade water. The reaction consisted of an initial denaturation step at 95°C for 3 min, 45 cycles of denaturation at 95°C for 10 seconds and annealing/extension at 62°C for 30 seconds. All results were normalised to the mean of the levels of both reference genes (*ACTB* and *HPRT1*) and relative quantification was calculated using a variation of the Livak method [26], described by Fleige and Pfaffl [27]. All samples were run in triplicate and the average values were calculated.

**Table 2:** Gene specific primers used for RT-qPCR.

Gene symbol	Full gene name	GenBank acession no.	TaqMan gene expression assay	Product size (bp)
*AQP1*	Aquaporin-1	NM_214454.1	Ss03385017_u1	97
*AQP3*	Aquaporin-3	NM_001110172.1	Ss03389620_m1	77
*AQP7*	Aquaporin-7	NM_001113438.1	Ss03389863_m1	86
*AQP9*	Aquaporin-9	NM_001112684.1	Ss03389741_m1	65
*AQP10*	Aquaporin-10	NM_001128454.1	Ss03374224_m1	61
*GK*	Glycerol kinase	NM_001143708.1	Ss03818509_m1	79
*mTOR*	Mechanistic target of rapamycin (serine/threonine kinase)	EU288086.1	Ss03377427_u1	82
*PIK3CG*	Phosphatidylinositol-4,5-bisphosphate 3-kinase, catalytic subunit gamma	NM_213939.1	Ss03392908_u1	68
***Housekeeping genes***				
*ACTB*	β-actin	XP_003124328.1	Ss03376081_u1	77
*HPRT1*	Hypoxanthine phosphoribosyltransferase 1	NM_001032376.2	Ss03388274_m1	73

### Preparation of membrane vesicles from the ileum portion of small intestine

Membrane vesicles were prepared from the ileum portion of small intestine samples by differential centrifugation with buffer without detergents. Approximately 6 g of ileum from each piglet was chopped into small pieces, removing visible endothelial tissue, and homogenised in mannitol-HEPES buffer (100 mM mannitol, 2 mM Tris-HEPES, pH 7.4) in a Waring blender for 2 × 1 min and then with a Potter-Elvehjem homogeniser. The homogenate was diluted with 10 mM MgCl_2_, gently mixed for 20 min and centrifuged at 3000 *g* for 20 min at 4°C in a Beckman Coulter Allegra 6R centrifuge. The supernatant was centrifuged at 30000 *g* for 30 min at 4°C in a Beckman 12–21 MIE centrifuge with a JA-20 rotor to obtain a pellet of crude membranes that was resuspended in a mannitol buffer (300 mM mannitol, 0.1 mM MgSO_4_, 0.02% NaN_3_ and 20 mM Tris/HEPES pH 7.4), transferred to a syringe and sheared by vigorously passing it 10 times through a 21-gauge needle and immediately used for transport experiments of total membranes. This protocol was adapted from other authors [28–30].

The protein content was determined by the BCA assay (Pierce™ BCA Protein Assay Kit, Thermo Scientific, MA, USA). The enrichment in specific activity of the apical markers, leucine-aminopeptidase and alkaline phosphatase (membrane vesicles/crude homogenate), assayed as previously described [31] was 9.41 ± 3.53 and 4.20 ± 2.16 (n = 20, mean ± SEM), respectively [32]. To obtain vesicles enriched in basolateral membranes (BLMV), 1 mL of the membrane suspension was loaded on a continuous linear sucrose gradient (25–55% (w/v) sucrose in 10 mM Tris-HEPES, pH 7.4), following the protocol described in [33]. The gradient was centrifuged at 100000 *g* for 60 min, the upper band was collected and resuspended in mannitol buffer and centrifuged at 100000 *g* for 30 min to remove traces of sucrose. The pellet was then resuspended in mannitol buffer by repeated passage through a 21-gauge needle, as above. The enrichment in basolateral membranes of the final preparation determined by the specific activity of the basolateral marker K^+^-phosphatase [34] was 11.75 ± 5.23 (n = 20, mean ± SEM). Membrane preparations obtained were either immediately used for experiments or stored at -20°C for later use. The vesicle size of all membrane preparations determined by the Quasi-Elastic Light Scattering (QELS) technique (Brookhaven Instruments BI-90, Holtsville, NY, USA), as described by Soveral et al. [31] revealed homogenous vesicles populations with 300 ± 21 nm diameter (mean ± SEM), regardless the dietary treatment.

### Membrane permeability of intestinal vesicles

Water and glycerol permeabilities of intestinal vesicles were evaluated by the stopped-flow technique (HI-TECH Scientific PQ/SF-53). Experiments were performed at 23°C for single measurements. Five replicates were analysed for each experimental condition. For the measurement of osmotic water permeability, membrane vesicles (0.2 mg protein/ml) resuspended in mannitol-HEPES buffer (300 mOsM) were mixed with an equal amount of isosmotic or hyperosmotic (500 mOsM) mannitol solutions to reach an inwardly directed gradient of the impermeant solute. The kinetics of vesicle shrinkage was measured from the time course of scattered light intensity at 400 nm until a stable light scatter signal was attained. The osmotic water permeability coefficient (P_f_) was estimated by fitting the light scatter signal to a single exponential curve and using the linear relation between P_f_ and the exponential time constant k [35], P_f_ = k (V_0_/A)(1/V_w_(osm_out_)_∞_), where V_w_ is the molar volume of water, V_0_/A is the initial volume to area ratio of the vesicle preparation, and (osm_out_)_∞_ is the final medium osmolarity after the applied osmotic gradient. For glycerol permeability, membrane vesicles equilibrated in 300 mOsM mannitol-HEPES buffer were challenged with a hyperosmotic glycerol solution (500 mOsM), creating an inwardly directed glycerol gradient. After the first fast vesicle shrinkage due to water outflow, glycerol influx in response to its chemical gradient was followed by water influx with subsequent vesicle reswelling. Glycerol permeability was calculated as P_gly_ = k (V_0_/A), where k is the single exponential time constant fitted to the light scattering signal of glycerol influx [36].

### Statistical analysis

Statistics was carried out with the Generalised Linear Mixed (GLM) model of Statistical Analysis System (SAS) software, version 9.4 (SAS Institute, Cary, NC, USA). Once normality was tested by Kolmogorov-Smirnov test and variance homogeneity, significant multiple comparisons test was carried out using the PDIFF option, adjusted with Tukey-Kramer, to determine statistical differences among dietary treatments. All data were presented as mean and standard error of the mean (SEM). A P value lower than 0.05 was considered statistically significant.

## Results

### Baseline characterisation of the dietary groups

Table 3 presents data on growth performance parameters and serum metabolites of piglets fed on amino acid-enriched diets, Gln and Cys, individually or combined. No variations were found for animal’s final body weight (P>0.05) as well as feed intake (P>0.05). Piglets fed on the mixture of glutamine and cystine had decreased concentrations of glucose (P<0.001), urea (P<0.001), creatinine (P<0.001), cholesterol (P<0.001), HDL-cholesterol (P<0.001), LDL-cholesterol (P<0.001), total protein (P = 0.006), total lipids (P<0.001), and phospholipids (P<0.001), relative to the other dietary treatments. The same pattern of variation was found for hepatic function markers, AST (P = 0.003), ALT (P<0.001), ALP (P<0.001) and GGT (P = 0.000). Conversely, piglets fed glutamine plus cystine had higher concentrations of VLDL-cholesterol (P = 0.001) and triacylglycerols (P = 0.001) than piglets fed on cystine and control diets. The redox status evaluation, through the assessment of total antioxidant capacity and glutathione peroxidase enzyme activity, was unchanged across dietary treatments (P = 0.146 and P = 0.838, respectively).

**Table 3:** Growth performance and serum biochemical metabolites of piglets fed on amino acid-enriched diets, glutamine and cystine, individually or combined.

	Control	Gln	Cys	Gln + Cys	SEM	P-value
*Growth performance*						
Initial body weight (g)	8.83	8.81	8.80	8.81	0.120	0.999
Final body weight (g)	23.4	22.9	22.6	22.5	0.400	0.303
Daily intake (g)	661	638	643	621	18.9	0.498
*Serum lipids*						
Total lipids (mg/dL)	334^bc^	344^c^	323^ab^	313^a^	3.12	< 0.001
Triacylglycerols (mg/dL)	42.8^a^	47.8^ab^	41.4^a^	52.4^b^	1.72	0.001
Phospholipids (ng/dL)	92.0^b^	97.0^c^	89.6^b^	79.0^a^	0.995	< 0.001
Cholesterol (mg/dL)	70.6^c^	73.2^c^	65.6^b^	55.4^a^	1.15	< 0.001
HDL-Chol (mg/dL)	30.0^c^	26.0^b^	28.4^bc^	22.4^a^	0.825	< 0.001
LDL-Chol (mg/dL)	32.0^c^	37.6^d^	28.9^b^	22.5^a^	0.481	< 0.001
VLDL-Chol (mg/dL)	8.56^a^	9.56^ab^	8.28^a^	10.5^b^	0.344	0.001
*Other serum metabolites*						
Glucose (mg/dL)	82.8^b^	96.6^c^	91.4^bc^	68.0^a^	2.69	< 0.001
Urea (mg/dL)	15.4^a^	20.8^b^	14.2^a^	13.0^a^	0.812	< 0.001
Creatinine (mg/dL)	0.676^b^	0.728^b^	0.736^b^	0.510^a^	0.026	< 0.001
Total protein (g/dL)	4.94^b^	5.00^b^	5.08^b^	4.20^a^	0.164	0.006
*Serum hepatic markers*						
ALT (U/L)	33.0^b^	40.2^c^	34.0^b^	24.6^a^	1.29	< 0.001
AST (U/L)	43.2^b^	44.8^b^	34.0^ab^	29.8^a^	2.70	0.003
ALP (U/L)	233^bc^	274^c^	224^b^	148^a^	12.0	< 0.001
GGT (U/L)	20.4^b^	22.8^b^	27.4^b^	10.4^a^	1.96	0.000
*Serum markers of redox status*						
Total antioxidant capacity (μmol/L)‡	158	162	161	186	8.92	0.146
Glutathione peroxidase activity (U/L)§	746	858	844	829	94.5	0.838

SEM, standard error of the mean

^a,b,c,d^Mean values within a row with unlike letters are significantly different (P<0.05)

‡Trolox equivalents

§One unit is the amount of Glutathione Peroxidase that produces 1 μmole of GSSG *per* min at room temperature and at pH 7.6.

### Glutamine and cystine, individually or combined, downregulate *AQP1*, *AQP7* and *AQP10* gene expression

**Fig 1:**
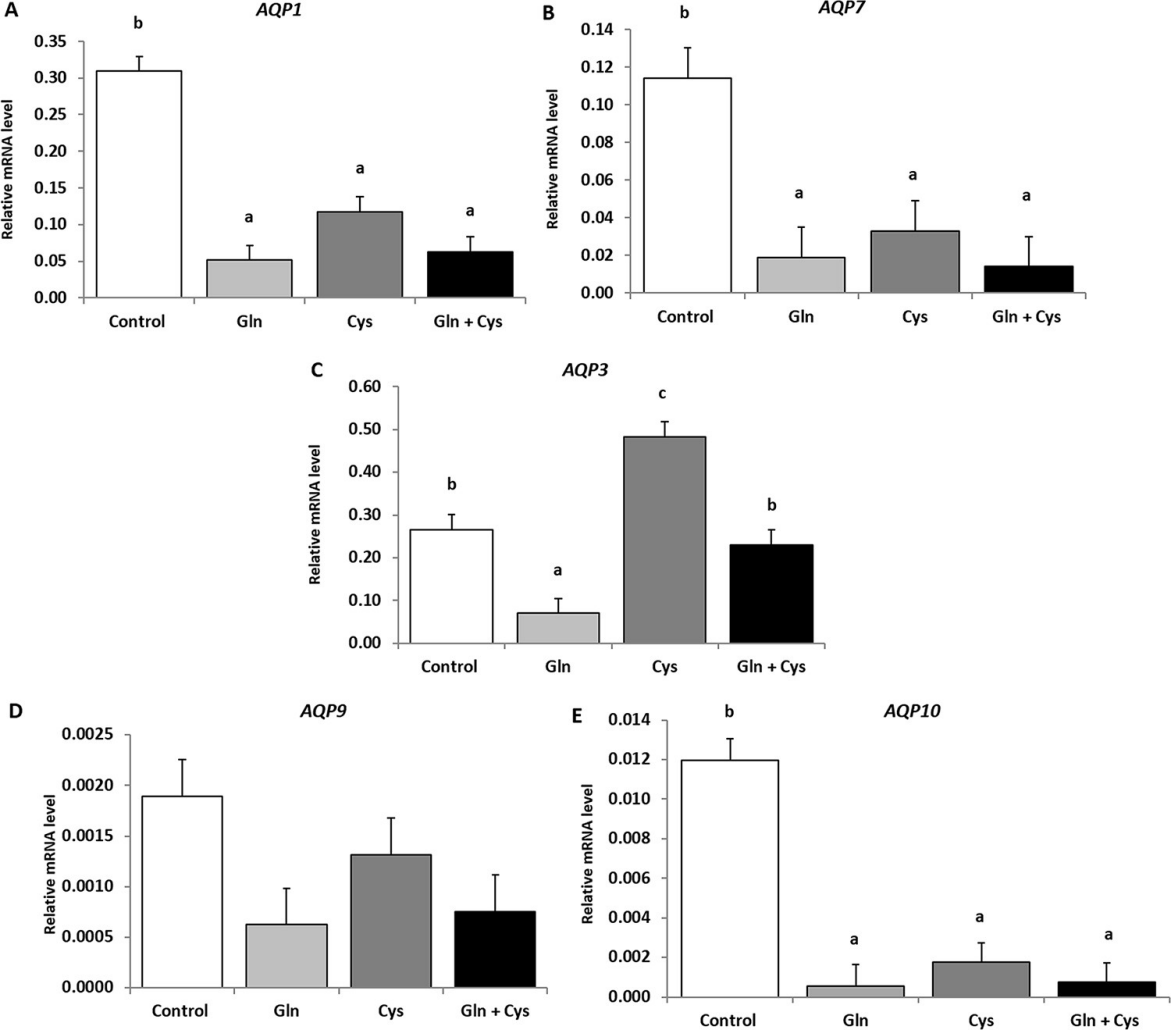
mRNA expression levels of *AQP1* (A), *AQP7* (B), *AQP3* (C), *AQP9* (D) and *AQP10* (E) in the ileum portion of the small intestine of piglets fed on amino acid-enriched diets, glutamine and cystine, individually or combined. Values are mean ± standard error of the mean (SEM) represented by vertical bars (n = 5). ^a,b,c^Mean values with unlike letters are significantly different (P<0.05).

Fig 1 presents the gene expression levels of the tested *AQPs* in the ileum portion of the small intestine of piglets fed on amino acid-enriched diets, glutamine and cystine, individually or combined. Comparing their transcriptional profile, *AQP3* mRNA levels were found predominant over the others (*AQP3*^a^ > *AQP1*^b^ > *AQP7*^c^, *AQP9*^c^, *AQP10*^c^), regardless the addition of amino acids (P<0.001). Moreover, a clear pattern of downregulation of *AQP1* (P<0.001) (Fig 1A), *AQP7* (P = 0.001) (Fig 1B) and *AQP10* (P<0.001) (Fig 1E) gene expression was found in piglets fed diets enriched in glutamine and cystine, individually or combined, relative to the control. The same trend was verified for *AQP9* mRNA levels, although not reaching statistical significance (P = 0.090) (Fig 1D). Cystine-enriched diet upregulated *AQP3* gene expression in relation to the other diets (P<0.001) (Fig 1C), but the combination of glutamine and cystine did not differ from the control (P>0.05) (Fig 1C).

### Cystine downregulates *GK* gene expression

**Fig 2:**
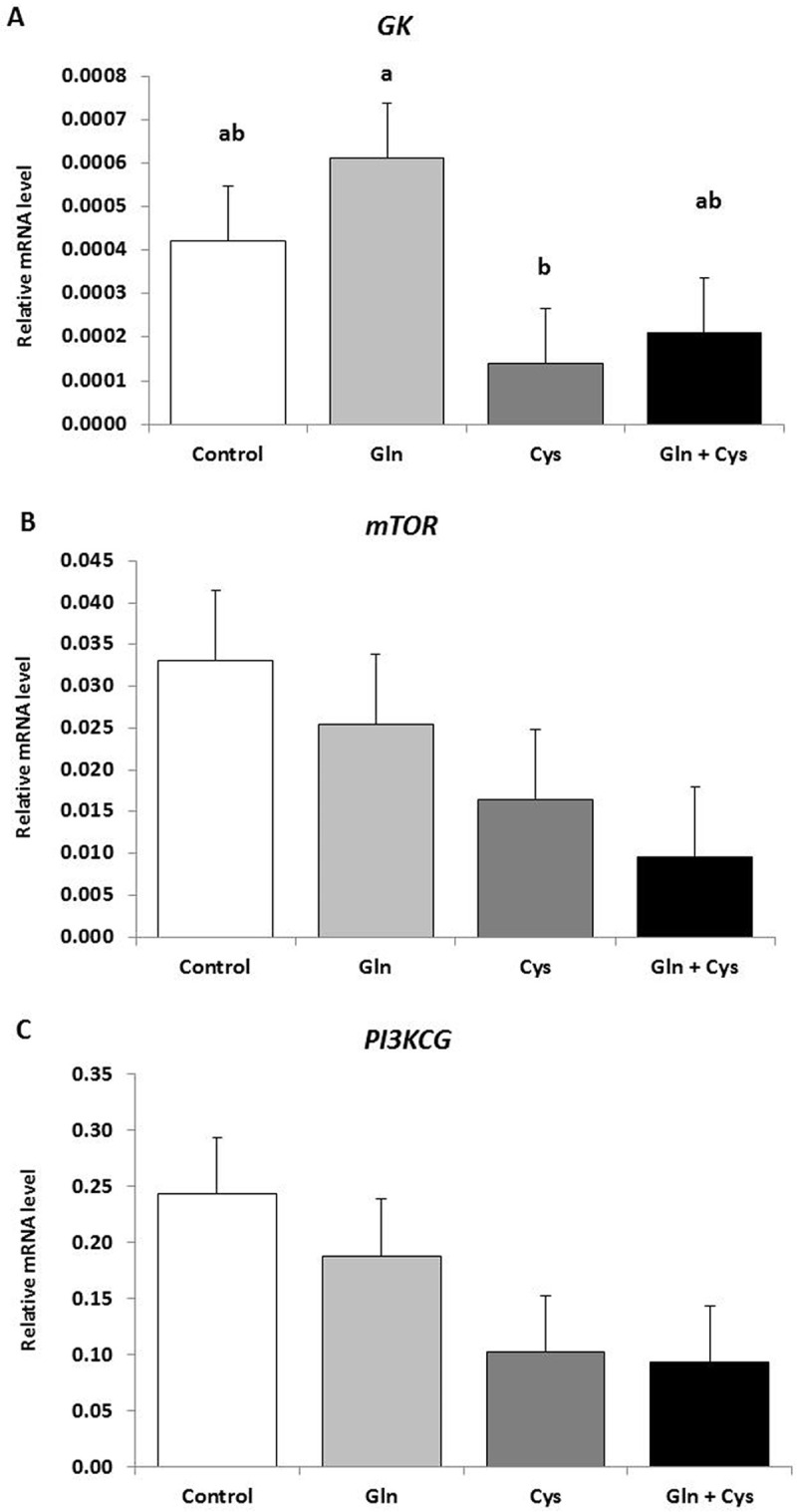
mRNA expression levels of *GK* (A), *mTOR* (B) and *PI3KCG* (C) in the ileum portion of the small intestine of piglets fed on amino acid-enriched diets, glutamine and cystine, individually or combined. Values are mean ± standard error of the mean (SEM) represented by vertical bars (n = 5). ^a,b^Mean values with unlike letters are significantly different (P<0.05).

Fig 2 presents the variation on gene expression levels of *GK* (A), *mTOR* (B) and *PI3KCG* (C) in the ileum portion of the small intestine of piglets fed glutamine and cystine, individually or combined. *GK* gene expression was downregulated by individually cystine relative to glutamine (P = 0.045) (Fig 2A). No further variations were found for *GK* mRNA levels among the other dietary treatments (P>0.05) (Fig 2A). The gene expression levels of *mTOR* (Fig 2B) and *PI3KCG* (Fig 2C) were kept unchanged by amino acid-enriched diets, glutamine and cystine, individually or combined (P = 0.206 and P = 0.119, respectively).

### Cystine increases glycerol permeability in intestinal vesicles enriched in basolateral membranes

**Fig 3:**
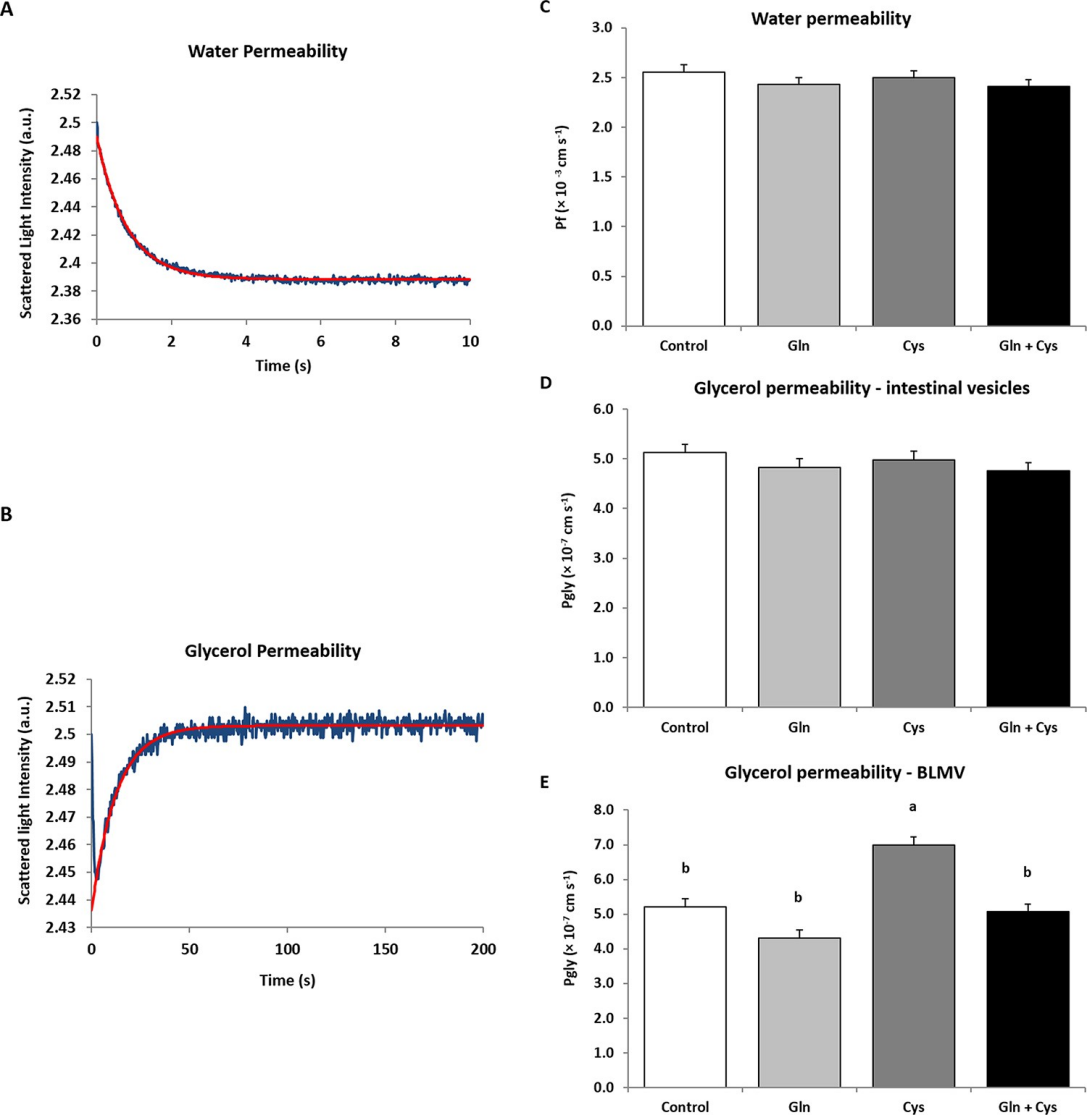
Representative stopped flow light scatter signals for evaluation of water permeability (P_f_) (A) and glycerol permeability (P_gly_) (B) of intestinal membrane vesicles, showing the time course of vesicle shrinkage (water outflux) or reswelling (glycerol influx) and the respective exponential fit used to calculate permeability. Effect of amino acid-enriched diets in glutamine, cystine and the combination of glutamine and cystine on P_f_ (C) and P_gly_ from intestinal vesicles (D) and from vesicles enriched in basolateral membranes (BLMV) (E) from the ileum portion of small intestine of piglets. Values are mean ± standard error of the mean (SEM) represented by vertical bars (n = 5). ^a,b^Mean values with unlike letters are significantly different (P<0.001).

To evaluate if the detected *AQPs* gene modulation by glutamine and cystine dietary treatments would impact on intestinal water or glycerol permeability across intestine, the ileum portion of the small intestine of piglets’ groups under study was used to prepare vesicles enriched in apical and in basolateral membranes. The vesicles from all dietary groups were assayed for water (P_f_) and glycerol (P_gly_) permeability. Fig 3A and 3B depict typical stopped flow signals showing the time course of vesicle volume shrinkage due to water outflux (Fig 3A) and vesicle reswelling due to glycerol influx (Fig 3B) with their respective exponential fits used to calculate P_f_ and P_gly_. Piglets fed on glutamine and cystine, individually or combined, displayed similar values of water permeability (P = 0.510) (Fig 3C) in both apical and basolateral membranes. Glycerol permeability was not different across dietary groups (P = 0.488) (Fig 3D) in vesicles enriched in apical membranes. However, in basolateral membranes, an increase in glycerol permeability was detected in piglets fed on cystine diet compared to the other dietary groups (P<0.001) (Fig 3E).

## Discussion

Ever since aquaporins were discovered, research on these transmembrane proteins has evolved providing knowledge on their pathophysiological importance [37]. AQPs were initially thought as bidirectional water-selective channels but they also play a central role in glycerol trafficking across the cell membrane barrier [11, 12] bridging tissues and organs, and contributing to energy homeostasis. The regulation of glycerol membrane permeation in the gastrointestinal tract is crucial to control fat deposition, lipolysis and gluconeogenesis. AQPs act as mediators of glycerol delivery underlying current investigation on molecular composition and regulation of intestinal barrier function. Therefore, targeting AQPs in the small intestine might turn into a novel approach to control metabolic disorders, such as insulin resistance, diabetes and obesity [38]. However, at present, scarce information is available, about the effects of nutrients, such as individual amino acids, on AQPs regulation in animal cells [39].

In this study, we used piglets fed glutamine and cystine-enriched diets to investigate the effects of supplemented amino acids on the general metabolic state of animals, through the assessment of growth performance and serum biochemical metabolites, followed by the modulation of *AQPs* gene expression and activity. The growth performance parameters of piglets, including daily feed intake, were not changed by amino acids supplementation. In terms of palatability, glutamine and cystine have been considered highly attractive in what taste properties concerns [40]. In fact, previous research has revealed that the supplementation of cystine and theanine (an analogue of glutamine) can increase appetite in rodents and humans [41, 42], contradicting our results and also, that the structure and function of the gut are beneficially preserved by glutamine [43].

For the biochemical profile, a consistent decreased pattern was found for glucose, cholesterol, HDL-cholesterol, LDL-cholesterol and total protein when pigs were fed the mixture of glutamine plus cystine. The impairment of total cholesterol and glucose at levels within normal range stands out as a promising result, thus suggesting the maintenance of glucose [44] and lipid metabolism [45] homeostasis. The same applies for total protein [46]. Urea and creatinine variations were indicative of unaffected renal function [47]. The systemic redox status was kept under balance in piglets from all dietary treatments. In fact, Wang et al. [48] reported that glutamine improves intestinal oxidative defence capacity. Cysteine (cystine is the oxidized dimer form of cysteine [49]) is generally the limiting amino acid for glutathione synthesis in humans [49–51], as in pigs [50], with recognised antioxidant properties [49] preventing many diseases, such as cancer, heart attack, stroke and diabetes [52]. In a larger scale trial using the same protocol, we revealed that the supplementation of glutamine and cystine improved total antioxidant capacity [53].

Regarding the gene expression of *AQPs* in piglets, the most expressed aquaporin isoform was *AQP3*, closely followed by *AQP1* and the remaining isoforms tested, regardless the dietary treatment. The magnitude and localization of the transcriptional profile found for these *AQPs* are in line with the literature [18, 38, 54–57]. Both amino acids, individually or combined, were responsible for a dramatic reduction on the transcriptional profile of *AQP1* (at least by 61.8%), *AQP7* (at least by 71.0%) and *AQP10* (at least by 88.8%). The same trend applies to *AQP9*, although not reaching statistical significance. However, the expected outcomes on reduction of water permeability were not verified in the ileum membranes from any of the piglets fed glutamine and cystine, individually or combined. In fact, our data shows that P_f_ was unaffected by diets. This finding contradicts other authors, who reported decreased permeability given by the integrity of the barrier, promoted by glutamine in an experimental mouse model [58]. Disparate results might be a consequence on the use of different animal models (piglets versus rodents), or the different technical approach used here to measure isolated membranes permeability. In fact, here we used a biophysical method to evaluate permeability of isolated membranes, while other studies have looked at the permeability of the whole tissue, which is totally dependent on the integrity of the epithelia. Although the relationship between gene, protein and function is not always directly proportional [59], we may also speculate that the higher intestinal permeability to water might occur upstream of the small intestine, at the level of duodenum or jejunum. Most importantly, the measured water permeability is the end result of the sum of two diffusional pathways, water channels and membrane phospholipid bilayer. Thus, the observed partial reduction at the transcriptional profile of *AQP1* promoted by glutamine and cystine is difficult to evaluate under the experimental conditions of this study and may have a minor impact on transepithelial permeability.

AQPs display differential distribution in apical and basolateral epithelial membranes, although their precise localization in intestinal epithelia is still controversial [18]. For instance, *AQP3* was found predominantly expressed in basolateral membranes of the small intestine [56, 60]. However, in other study comparing normal subjects vs. celiac disease patients, *AQP3* was abundantly expressed but the protein localized at enterocyte apical membranes [16]. Discrepancies in results were attributed to differences in used antibodies, tissue sampling procedures and conservation, patient condition (fasted or fed), and/or other methodological details. Moreover, in pathological conditions, AQPs localization may shift from apical to basolateral membranes, as observed for AQP7 in colon inflammatory bowel disease patients [15]. In this study, we found AQP3 as the most abundantly expressed in piglets’ ileum, in agreement with rodents and humans [18, 56] where AQP3 is predominantly located in basolateral membranes. Using intestinal membrane vesicles enriched in basolateral membranes, we were able to ascertain the impact of *AQP3* transcript upregulation by dietary cystine supplementation on intestinal glycerol permeability. Inversely, although AQP7 and AQP10 are described as predominantly located in apical membranes [56, 61], significant changes in their much lower transcript level compared to *AQP3* may result in undetectable changes on glycerol permeability induced by these amino acid-enriched diets.

The interplay between AQPs has been reported previously in human obesity. AQP3 and AQP7 have been described to modulate glycerol efflux from adipose tissue, thus controlling the glycerol influx into hepatocytes, via AQP9 to prevent the excessive lipid accumulation and the subsequent aggravation of hyperglycemia [21]. While AQP3 upregulation by cystine supplementation may suggest facilitated glycerol absorption from the intestinal lumen favoring triacylglycerols synthesis and lipid accumulation in the adipose tissue, the downregulation of *AQP3* by glutamine-enriched diet shown here (at least by 69.4%) might be a promising strategy to limit the excess dietary glycerol entrance into the bloodstream. Therefore, these data suggest that intestinal AQP3 might also participate in the interplay between adipose AQP7 and hepatic AQP9 to maintain body energy homeostasis.

Glycerol kinase plays a central role in adipogenesis and gluconeogenesis promoting the modification of glycerol in its biological active form, glycerol-3-phosphate [62]. Herein, the low mRNA levels of *GK* detected in the ileum of piglets are justified by tissue specificity, as glycerol kinase is highly expressed in liver, kidney and testis [63], rather than in the small intestine. However, it is interesting to notice that glutamine-enriched diet increased *GK* expression (at least by 77.0%) in relation to cystine-enriched diet leaving this molecule available to be used in the enterocyte metabolism, simultaneously with the impairment of *AQP3* expression, the main route for dietary glycerol to reach the bloodstream. In turn, PI3K/Akt/mTOR is a signaling pathway regulating *AQP3* expression in different metabolic events [21, 39, 64]. Our data presented no variations on gene expression levels of *mTOR* and *PI3KCG* by diets and consequently, no association between glycerol channels and these intracellular mediators was accomplished in the small intestine of piglets, under these experimental conditions.

## Conclusions

The integrative methodological approach applied in this study using the ileum portion of the small intestine of piglets unveils *AQPs* gene regulation by dietary amino acids, thus providing a novel insight into the molecular players involved in gut physiology. A clear influence on the transcriptional profile of the water channel *AQP1* and aquaglyceroporins *AQP7* and *AQP10* by glutamine and cystine, individually or combined, was observed, although their impact on the total water movements might be masked by a larger contribution of the lipid bilayer diffusional pathway. However, upregulation of *AQP3* gene together with increased glycerol permeability of intestinal basolateral membranes, suggest that glycerol intestinal reabsorption via intestinal AQP3 might be favored in piglets fed cystine diet, revealing AQP3 as a new player in fat accumulation. Altogether, our data show that amino acids dietary supplementation can modulate intestinal AQPs expression and unveil AQP3 as a promising target in adipogenesis regulation. Due to the similarities between pigs and humans, this study allows the translation of porcine data to humans and warrants further additional research to validate its findings.

## Abbreviations

ACTB: β-actin
AQP: aquaporin
BLMV: basolateral membrane vesicles
GK: glycerol kinase
HPRT1: hypoxanthine phosphoribosyltransferase 1
mTOR: mammalian target of rapamycin
PIK3CG: phosphatidylinositol-4,5-bisphosphate 3-kinase, catalytic subunit gamma
